# Does surface completion fail to support uncrowding?

**DOI:** 10.1167/jov.25.3.1

**Published:** 2025-03-03

**Authors:** Lisa Schwetlick, Mauro Manassi, Michael H. Herzog, Gregory Francis

**Affiliations:** 1 Laboratory of Psychophysics, Brain Mind Institute, École Polytechnique Fédérale de Lausanne (EPFL), Lausanne, Switzerland; 2 Laboratoire des Systèmes Perceptifs (UMR8248) École normale supérieure, Paris, France; 3 School of Psychology, University of Aberdeen, Aberdeen, UK; 4 Department of Psychological Sciences, Purdue University, West Lafayette, IN, USA

**Keywords:** crowding, mid-level vision, modeling

## Abstract

In crowding, perception of a target deteriorates in the presence of nearby elements. As the entire stimulus configuration across large parts of the visual field influences crowding and not just nearby elements, low-level explanations, such as local pooling, do not suffice. To explain the effects of stimulus configuration, grouping was proposed as the key, and we implemented these ideas in a neural network model (LAMINART). In a recent publication, Moore and Zheng (2024) used a set of stimuli designed to induce surface completion cues, such as occlusion, and found that they had no effect on crowding. Based on these results, the authors questioned the role of grouping in crowding. Here we show that the stimuli Moore and Zheng used do not induce the intended perceptual occlusion effects. Hence, their conclusions are not warranted. Additionally, simulations of the LAMINART model explain the results of Moore and Zheng with the existing model characteristics.

## Introduction

**Figure 1.**
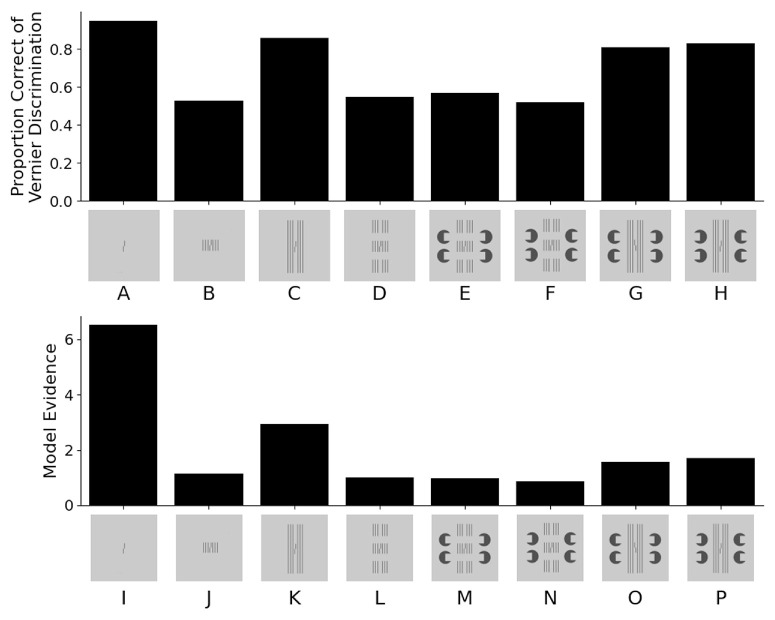
The top panel (conditions A–H) shows experimental results from Moore and Zheng (2024). (A) In a vernier discrimination task, observers were asked to discriminate the offset direction of the lower bar with respect to the upper bar (left vs. right). (B) The proportion of correct responses strongly decreases (crowding) when flanking lines of the same length are presented with the target. (C) Longer flanking lines lead to weaker crowding (uncrowding). (D–H) The novel stimuli used in the study. (D) Disrupting the long lines by introducing gaps leads to stronger crowding (gapped flankers). (E) Moore and Zheng (2024) expected to find performance similar to the long lines condition because, as they propose, the flankers appear as a long grating occluded by horizonal bars induced by the four Pacmans. Conditions (F), (G), and (H) served as control conditions. However, performance in condition (E) is no different than in conditions (D) and (F), which share the gaps but do not have a virtual occluder. The bottom panel shows the performance of the LAMINART model for the same stimuli. The y-axis shows the model evidence for a right vernier (calculated as a difference of template matches for left and right verniers). The bottom panel (I–P) shows the model evidence for the same stimuli as above. Note that the model are qualitatively comparable to the experimental results.

In crowding, perception of a target deteriorates when neighboring elements are added. Crowding is ubiquitous in natural vision since stimuli are rarely presented in isolation (except, perhaps, during psychophysics experiments). Classically, crowding was explained by local interactions that pooled information of the target and the flankers and thereby lose target information. However, such simple pooling models do not provide a comprehensive framework for understanding visual crowding since more recent research shows that the entire visual scene determines crowding and not only nearby elements (Bornet, Doerig, Herzog, Francis, & Van der Burg, 2021; Doerig et al., 2019; Herzog, Sayim, Chicherov, & Manassi, 2015; Herzog & Manassi, 2015; Manassi, Sayim, & Herzog, 2012; Manassi, Hermens, Francis, & Herzog, 2015; Manassi, Lonchampt, Clarke, & Herzog, 2016). A clear-cut example is uncrowding, where the addition of elements to an already crowded target improves performance. For example, in Figure 1, vernier offset discrimination strongly deteriorates when flanking lines are presented next to the vernier (Figures 1A vs. 1B) (i.e., crowding). When these lines are replaced by longer lines (Figures 1B vs. 1C), crowding diminishes, even though there is more flanker “signal.” Crowding further diminishes when more long flanking lines are presented, approaching performance in the unflanked, vernier-only condition (Herzog et al., 2015; Moore & Zheng, 2024).

Consequently, uncrowding challenges models in which flankers deteriorate performance because of *locally* added noise, increased lateral inhibition, or similar mechanisms. To account for these results, we proposed a two-stage model, in which, first, elements in a scene are grouped into separate objects and textures. Second, interference occurs with respect to the perceived grouping: Crowding occurs within grouped elements and not by, for example, pooling over small inflexible regions defined by the receptive field size of neurons.

Importantly, our hypothesis of grouping as a central mechanism for crowding is a perceptual hypothesis and, in this respect, is almost trivial or tautological: If the target is perceived to group with the flankers, there is crowding. If the perception of the target is perceived as separate, there is no crowding. Indeed, there are significant correlations between crowding and subjective ratings of how much the target stands out from the flankers (Malania, Herzog, & Westheimer, 2007; Manassi et al., 2012; Saarela, Sayim, Westheimer, & Herzog, 2009; Wolford & Chambers, 1983). The subjective perception of the stimulus configuration, therefore, is critical (Herzog et al., 2015) and must be tested independently, in addition to performance with accuracy measures.

Perceptual grouping of elements is strongly influenced by three-dimensional (3D) information such as occlusion. In a recent study, Moore and Zheng (2024) aimed to investigate whether mid-level mediation can induce ungrouping of the flankers from a vernier target, thus provoking uncrowding. They presented three separate short vertical gratings stacked vertically, with the middle one containing the vernier target (see Figure 1D). As expected, crowding was strong because the vernier grouped with the elements of the central grating. Next, they added Pacmans to induce occlusion cues. They suggested that this change created the percept of a long grating behind horizontal bars (see Figure 1E), which should lead to uncrowding (as in Figure 1C). Under the same assumptions, perceiving the lines as three separate gratings with no induced horizontal bar (as in Figure 1D) would lead to crowding. Moore and Zheng (2024) found evidence only for crowding. They suggest, in line with low-level explanations of crowding, that the strong crowding comes from the line terminators at the grating endings and that the long lines in Figure 1C simply “mask” the low-level terminators by continuing the lines. They suggest that similar “hidden” low-level cues may be in operation in the many other demonstrations where grouping was proposed to be key and, hence, question the grouping account in general.

Based on their findings, Moore and Zheng (2024) conclude that midlevel mediation, such as 3D cues, does not significantly contribute to visual crowding in their experiment. While their title, “Limited Midlevel Mediation of Visual Crowding: Surface Completion Fails to Support Uncrowding,” suggests a broader critique of midlevel mediation, their conclusions in the text are explicitly confined to the specific uncrowding effect they tested. Moreover, Moore and Zheng (2024) highlight several instances where the LAMINART model, an existing (mid-level model) of visual perception, failed to predict human performance in crowding tasks and conclude that LAMINART’s account, including its use of recurrent architecture and global influences, is insufficient to explain crowding in complex displays. While no model is perfect, we demonstrate that LAMINART can explain their experimental results based on connections formed between grouped elements without a need to consider occlusions.

The main conclusion of Moore and Zheng (2024) depends on the assumption that the Pacmans induce occlusion cues that lead to the percept of a long grating occluded by two horizontal bars. While we agree that when viewed foveally for long durations and without a specific task, the stimuli induce those 3D occlusion cues, here, we empirically show that their stimuli, when presented in the periphery, do not seem to generate this percept. It is more likely that the occluders effectively function as additional distractors, particularly because they appear between the fixation point and the gratings.

## Rating experiment

In order to quantify the subjective perception of the stimuli used by Moore and Zheng (2024), we conducted a rating experiment. Participants were paid students of the École Polytechnique Fédérale de Lausanne (EPFL) between the ages of 19 and 24. Fifteen participants with normal or corrected-to-normal vision, measured with the Freiburg Visual Acuity Test (Bach, 1996), were recruited for the study. All participants provided informed consent before participating. All protocols conformed to Standard 8 of the American Psychological Associations Ethical Principles of Psychologists and Code of Conduct (2010) and to the Declaration of Helsinki (with the exception of Article 35 concerning preregistration in a public database). Observers were informed that they could withdraw from the experiment at any time without penalty.

We presented the vernier with six flanker configurations: long flankers (Figure 1C), gapped flankers (Figure 1D), gapped flankers with inside-facing inducers (Figure 1E), long flankers with inside-facing inducers (Figure 1G), long flankers with outside-facing Pacmans (Figure 1H), and gapped flankers with outside-facing Pacmans (Figure 1F).

Just as in Experiment 5 in the original study (Moore & Zheng, 2024), the stimulus was presented at a screen distance of 55 cm. A vernier was presented at an eccentricity of 10.5 degrees and had a length of 1.8° and a width of 210 arcseconds. The vernier offset was 0.2°. The vernier was surrounded by three flanker lines on the left and the right. The short flankers had the same height as the vernier, with the lines spaced 0.4° apart. Long flankers were the same except their height was 8.5°. Gapped stimuli were identical to the long flankers but included a gap that was aligned with the top and bottom of the vernier and had a height of 1.25°. The Pacman inducers were aligned with the gap, at a horizontal distance of 3.33° to the vernier (Moore and Zheng [2024] slightly varied this distance across their five experiments; our value is close to what they used). The background was a very light gray (75% luminance), and each inducer was a dark gray (25% luminance) circle with radius 1.125° and a cutout horizontally from the center to the circle's perimeter and same height as the gap. The stimulus remained on the screen until the participant responded, as in the original Experiment 5, allowing participants to sample the peripheral display for as long as they required to make their judgment.

Participants were explicitly instructed to maintain fixation on the central fixation point throughout the experiment and to avoid moving their eyes to directly view the peripheral target. These instructions emphasized that the task was about subjective perception in the visual periphery. Participants were reminded throughout the session to prioritize fixation. The exact instruction text can be found in the Appendix.

First, participants judged whether the bottom line of the vernier was offset to the left or right using the arrow keys on the keyboard. Following the vernier task, participants were asked to judge the flanker gratings to quantify the subjective perceptions of the stimuli. Specifically, they were asked to rank their perception (“Did you see 3 separate gratings or a single grating, partially covered by horizontal bars?”) on a Likert scale from 1 to 5, with 1 indicating “I saw a single grating” and 5 indicating “I saw 3 completely separate gratings.” In order to ensure the participants understood the task, the first instructions introduced them to the concept of illusory overlap and long versus three separate gratings (see Appendix).

The key question was whether participants perceived a continuous, occluded grating or a gapped grating. To address this directly, we asked participants to report their subjective perception directly, rather than using an indirect (objective) measure. While psychophysics experiments often assess perceptual differences through measures like discrimination ability (Moore & Egeth, 1997; Moore, Grosjean, & Lleras, 2003; Pomerantz, Sager, & Stoever, 1977), subjective ratings can provide direct insights into perceptual experience. Notably, subjective and objective measures frequently converge in their characterization of phenomena such as illusory contours (Francis & Wede, 2010).

Each flanker configuration was shown twice on each side (left and right), and the presentation order of both the configurations and the left/right side was randomized for each participant to prevent order effects. This setup yields a total of four ratings per configuration and a total of 24 judgments.

**Figure 2.**
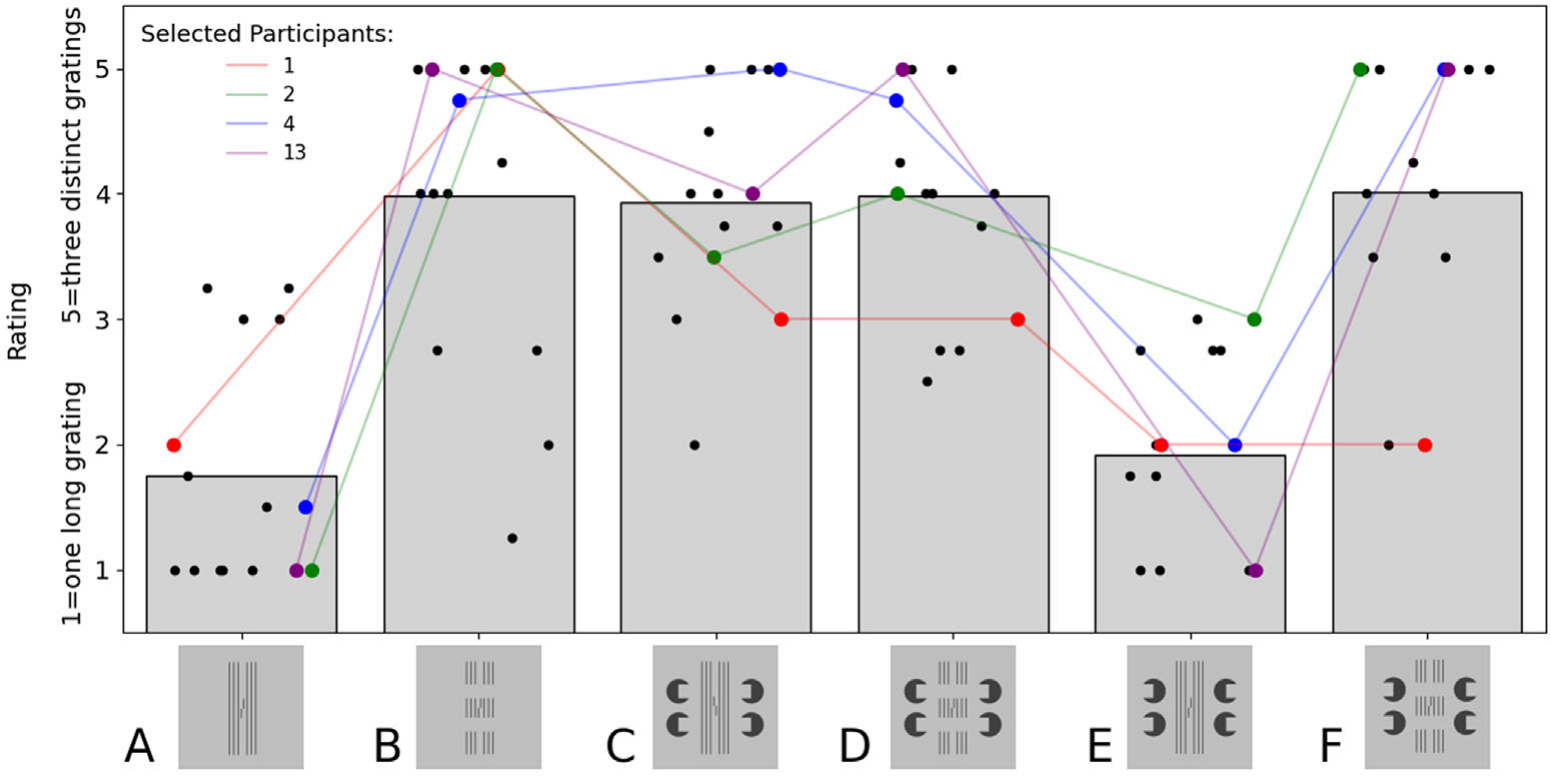
Results of the rating task for conditions A to F. The ratings were given on a scale of 1 to 5, where 1 indicated that participants saw one long grating and 5 indicated that they saw three completely distinct gratings. The bars indicate the average of all ratings. Black dots are mean ratings for individual participants. The thinner colored lines show the ratings of four randomly selected participants, where each data point is the average of four trials, collapsed over the two left and two right presentations.

Figure 2 plots the mean rating of the flanker gratings for each configuration. The long flanker condition (A) leads to a significantly lower rating than the gapped flanker inward-facing Pacman condition, which is intended to produce an illusory overlaid bar (D). In direct contradiction to the interpretation of the flanker elements forming one long grating behind occluding horizontal bars, this stimulus is generally interpreted as three separate gratings. We performed a Wilcoxon signed rank test between the ratings of the long flankers and the ratings of the gapped flanker inward-facing condition and found a significant difference between the groups (sum of the ranks = 12.5, *p* = 0.001). In general, all the gapped flanker conditions (B, D, F) consistently have an average rating of about 4, where a value of 5 indicates a percept of three distinct gratings. This result suggests that the stimuli in Moore and Zheng (2024) did not induce the intended occlusion percepts.

Conditions E and F (i.e., the conditions with outward-facing Pacmans) are rated on average, as expected: The gapped flankers in condition F are seen as distinct gratings, and the long flankers in condition E are seen as long. Strikingly, the inward-facing Pacmans, aside from not inducing the intended long-flanker percept (D), also seem to generally cause uncertainty about the flanking lines. Condition C received the same average rating as condition D, although, foveally, it is clear that the flankers in (C) are uninterrupted. The inward-facing Pacmans seem to evoke gap cues that interact with the line flankers.

Finally, we note that there was a large amount of variability even within participants (see Appendix for the ratings of all participants). The mean difference in ratings between trials of the same condition by the same person is 1.94. In other words, a participant who responded with 5 (“3 completely distinct gratings”) on one trial might answer 3 (“neutral”) on another trial. This inconsistency indicates, again, that the stimuli do not reliably induce the intended occlusion percepts or, indeed, any consistent percepts at all. If viewed foveally, for example, if participants had not maintained fixation as instructed, we would expect no such inconsistencies. Hence, the stimuli of Moore and Zheng (2024) did, first, not adequately induce the intended occlusion effects (which is a necessary premise for the experiments) and, second, seem to lead to rather unstable percepts in general.

## Modeling

If the stimuli used by Moore and Zheng (2024) did not induce the intended occlusion percepts, then it makes sense to consider whether mid-level models without occlusion can account for their findings. We focus on the LAMINART model that was used in Francis, Manassi, and Herzog (2017) to explain a variety of uncrowding effects.

**Figure 3.**
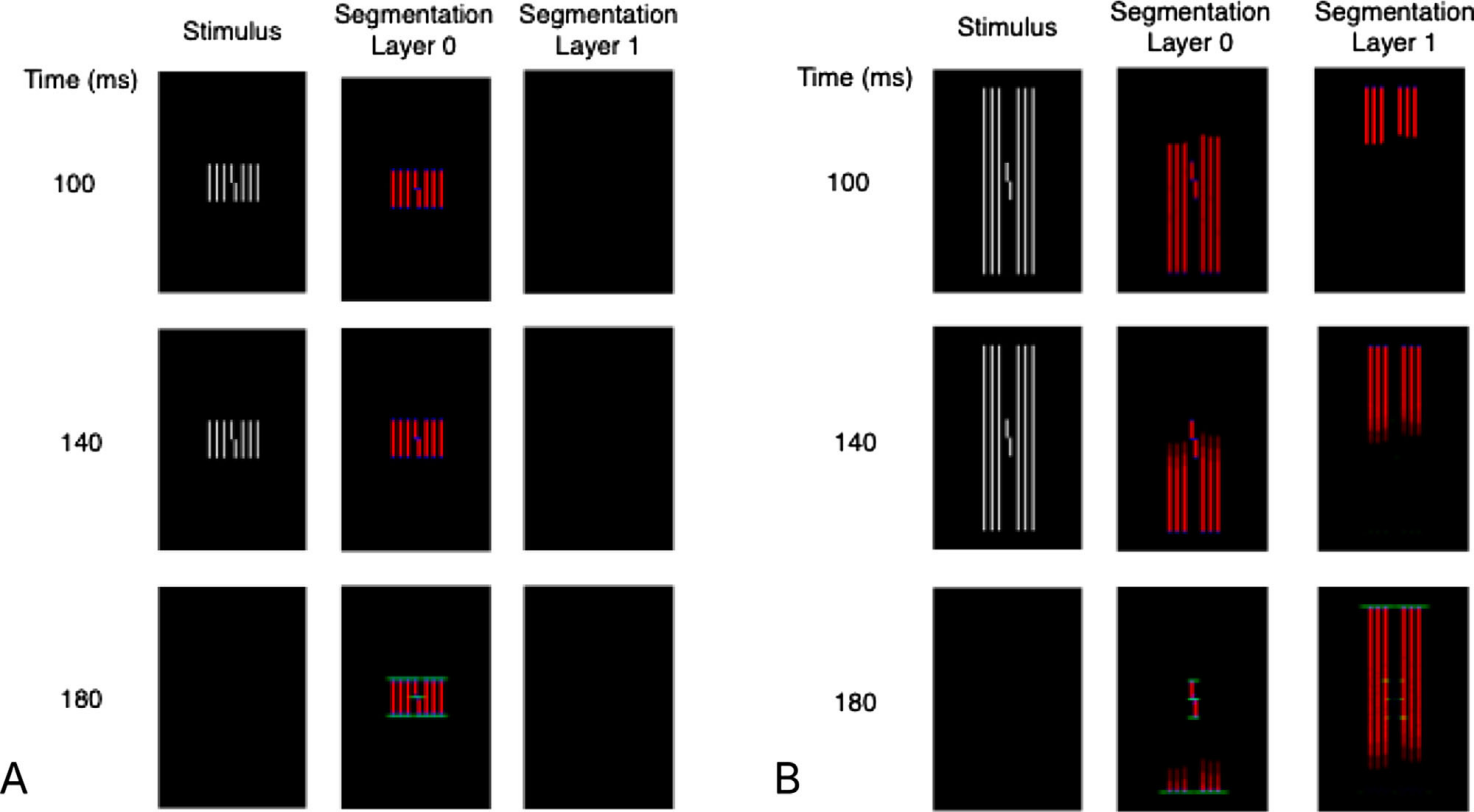
(A) Model simulations of a vernier with equal length flankers. The green pixels along the top and bottom of the flanker contours indicate grouping connections between the display elements. (B) Model simulations of a vernier with long flankers. Top-down selection signals placed at the top of the image spread across the flanker contours and shift their representation to Segmentation Layer 1. This segmentation process leaves the vernier mostly isolated in Segmentation Layer 0, thereby leading to good discrimination.

The LAMINART model emulates neural circuits in visual cortex using integrate-and-fire neurons. The model’s explanation of uncrowding involves two key mid-level properties: contour grouping and segmentation. Contour grouping refers to an interpolation process that links together oriented edges that are aligned in the same orientation. For example, Figure 3A shows activity for the model's orientation-selective complex cells in area V2 of visual cortex (for reasons explained below, we call this model part Segmentation Layer 0) at various times during a model simulation. A red pixel indicates strong activity (high firing rate) for vertically tuned cells, and a green pixel indicates strong activity for horizontally tuned cells. (Some pixels have blue activity, which indicates strong activity for a diagonally oriented cell, but these cells hardly play a role for the stimuli considered here.) The numbers in the leftmost column of Figure 3A indicate simulation time. The images show the accumulated spike counts at each pixel over the preceding 20 milliseconds of simulated time. As in Experiments 1 to 4 in the original article (Moore & Zheng, 2024), the stimulus was presented for 120 milliseconds (onset at time 20 and offset at time 140).

It takes time for information about the stimulus to reach the cells in area V2, but by time 100 milliseconds, strong responses form among vertically sensitive neurons along the contour of each vertical stimulus line. Weak responses also form (not visible) among horizontally sensitive neurons along the top and bottom of each vertical line. Visual processing in the model continues beyond stimulus offset (Francis, Grossberg, & Mingolla, 1994; Francis, 1997), and grouping among these persisting contour signals is indicated by the connected green pixels along the top and bottom of the model's representation of the stimulus contours. These connections are similar to illusory contours (Grossberg & Mingolla, 1985a, Grossberg & Mingolla, 1985b; Kon & Francis, 2022). Critically, the contours that correspond to the target vernier are connected to the contours that correspond to the flanking lines, thus indicating that the vernier and the flankers are part of a common group. Crowding is strong for this stimulus because a template designed to discriminate between a right vernier (as shown) and a left vernier also pools information from the flankers. Figures 1I–P quantify the model evidence (a comparison of left and right shift templates) for various stimuli. Larger numbers correspond to a higher proportion correct.

An important impact of contour grouping is realized in the second model property: segmentation. Top-down selection signals spread across connected contours and shift those contours from a default Segmentation Layer 0 into a separate representation (Segmentation Layer 1). Selective placement of the top-down selection signals can modify the representation of visual information so that the template for vernier discrimination is mostly unaffected by the flankers. For example, Figure 3B shows model behavior in response to the long flankers condition. The top-down selection signals are a pair of circles that (roughly) cover the top parts of the left and right flankers. The selection signals spread down the flanker contours and shift the contour signals at each selected pixel into Segmentation Layer 1. It takes almost 100 milliseconds for the shift to complete, but near the end of the process, the contours that correspond to the vernier are almost isolated in Segmentation Layer 0. When the template calculations are performed on the signals in Segmentation Layer 0, it is almost as if the vernier were presented by itself for part of the simulation. The separation of the flankers from the vernier is what leads to uncrowding (details are in Francis et al., 2017), as quantitatively shown in Figures 1I–P.

**Figure 4.**
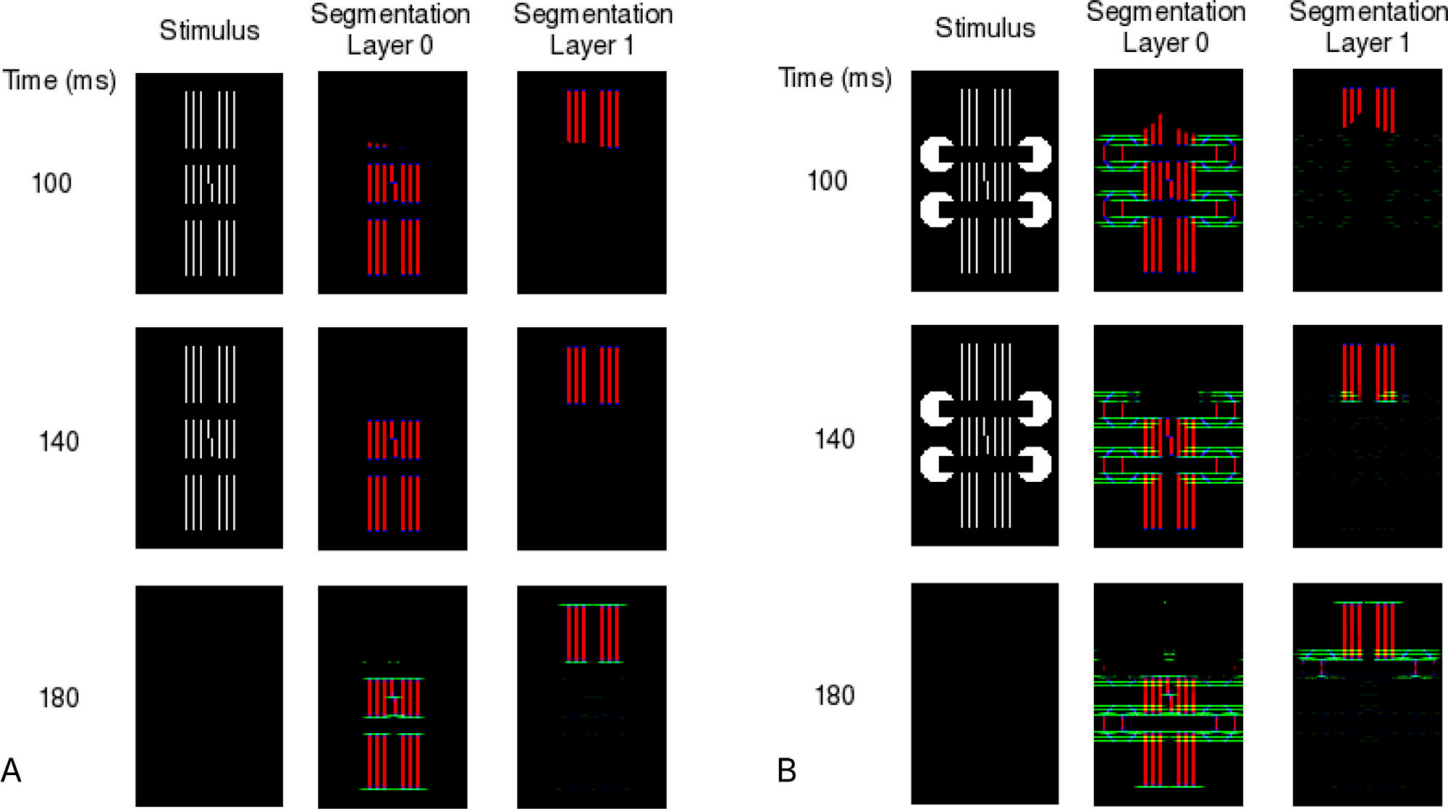
(A) Model simulations of a vernier with long flankers having a gap. The selection signals spread only across the contours at the top of the flankers. The vernier remains close to the flanker middles and thereby is crowded. (B) Model simulations of the long flankers having a gap and Pacman inducers. Again, the selection signals do not separate the vernier target and flanking elements.

Figure 4A shows that when the long flankers include a gap, the model forms connections between the vernier and the middle parts of the flankers, which prevents the segmentation of the vernier and flanker elements. Rather than selecting all of the flanking elements, the selection signal only spreads across the top elements. Without segmentation of the flanking elements next to the vernier, the vernier is crowded by those flankers.

The story is much the same for the other stimuli used by Moore and Zheng (2024). Figure 4B shows similar simulation results when the long flankers include a gap and inducing elements. (Simulation code and results for all stimuli are available at the Open Science Framework.) The added inducers do not alter (and sometimes even strengthen) the horizontal connections between the middle parts of the flankers and the vernier, and they do not allow the segmentation process to separate the vernier from the nearby flanking elements. The model simulations predict that the extra inducers hardly affect performance at all (Figures 1M–P), which is what Moore and Zheng (2024) reported. The correlation between the empirical proportion correct scores and the model evidence scores is *r* = 0.77.

Taken all together, the findings in Moore and Zheng (2024) are predicted by mid-level models such as LAMINART, which implement mechanisms of contour grouping and segmentation.

## Conclusions

Classic explanations of crowding focus on local neural interactions between the target and the neighboring flankers in accordance with findings that flankers outside Bouma's window do not contribute to crowding. However, uncrowding has shown that elements in large parts of the visual field can dramatically change crowding (Figure 1). We have argued that grouping is key: When the target ungroups from the flankers, crowding is weak. Moore and Zheng (2024) concluded that their findings do not provide strong evidence for mid-level mediation of the specific uncrowding effect tested and suggested that low-level explanations remain viable.

Specifically, they argued that the Pacmans in Figure 1 induce strong mid-level occlusion cues, which lead to the percept of a long grating occluded by two bright bars and hence should lead to uncrowding. Since they did not find this predicted effect, they concluded that mid-level processing, related to occlusion and figure completion, seems not to play a role, thereby defeating the grouping explanation. Here, we challenged their conclusions on three grounds: (1) Contrary to their assumption, Pacmans did not induce the intended occlusion cues; (2) substantial evidence in the literature supports mid-level mediation, with low-level features alone being insufficient for explaining uncrowding; and (3) the LAMINART model accurately replicates their results with grouping mechanisms that would be treated as mid-level mediation.

The Moore and Zheng (2024) stimuli implicitly assumed that the Pacmans in Figure 1 induce a 3D percept of horizontal illusory bars overlaying the long flanker grating. However, the ratings in our experiment suggest that the Pacmans did not produce the intended effect. In addition, the ratings varied strongly, even within individual participants and even for unambiguous conditions (e.g., long lines, Figure 2B). These observations cast doubt on whether such complex flanker configurations, viewed in the periphery, are fully assembled at all or interpreted and used as grouping cues. It seems more likely that there is only partial assembly of the cues, leading to a tendency to prefer simple, non-3D interpretations. Although Maertens and Pollmann (2007) showed that subjects can perceive illusory contours in the periphery, their participants underwent “extensive training” before data could be collected and the phenomenon is not common. In foveal vision, it is known that stereo cues reduce crowding very strongly (Astle, McGovern, & McGraw, 2014; Sayim, Westheimer, & Herzog, 2008). Peripheral sampling often obscures the image features required to define depth relations, such as fine disparity cues. Thus, we agree that it should, in principle, be possible to induce a release from crowding using occlusion and 3D perception using a study design with true binocular stereo cues. However, it is entirely possible that binocular vision is limited in the periphery and that without it, mid-level processes like depth-based surface representation may not function effectively.

**Figure 5.**
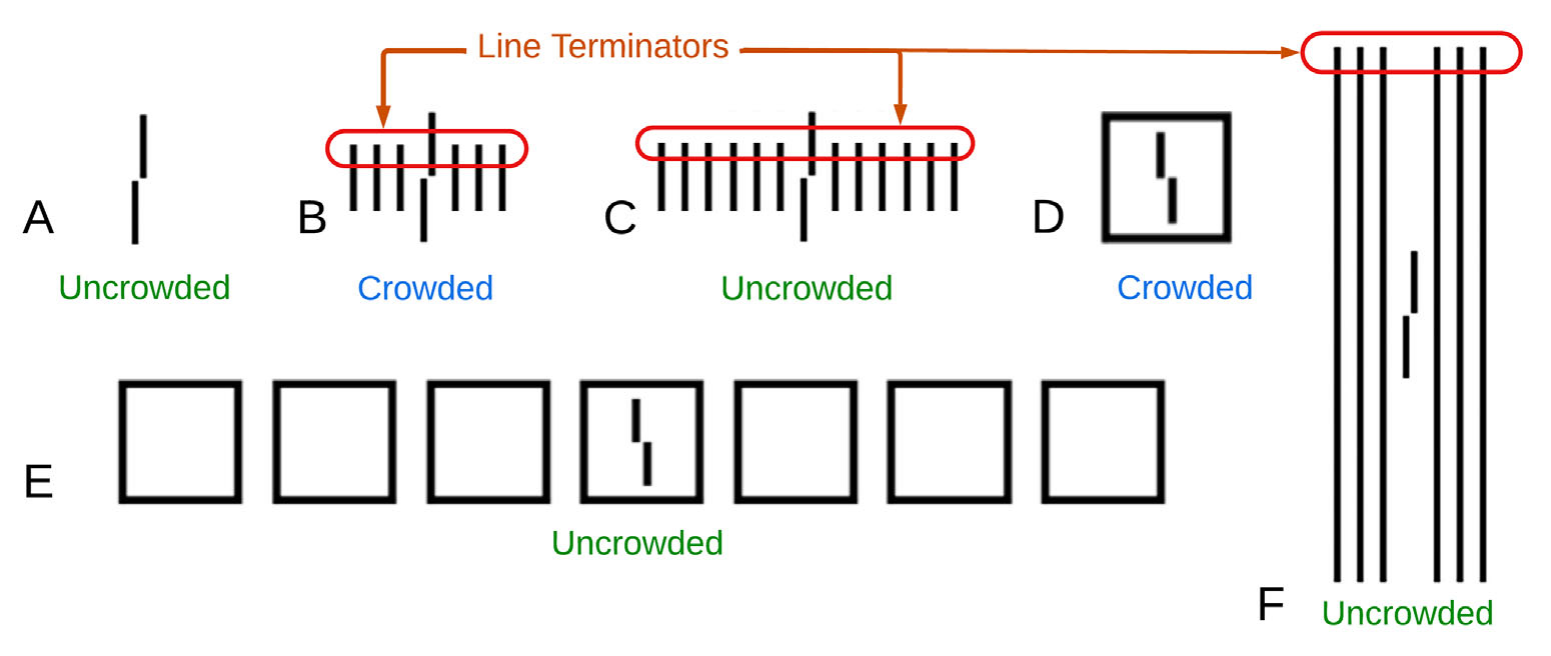
Line terminators are not sufficient to explain uncrowding. (A) An uncrowded vernier. Given that few short lines (B) cause crowding and long lines (F) cause uncrowding, it is possible that line terminators are causing the interference. However, adding more short lines (C), and therewith more line terminators, causes uncrowding. Furthermore, the square does not have line terminators at all and still induces crowding (D), and many squares induce uncrowding (E).

In contrast to the emphasis Moore and Zheng (2024) placed on the importance of low-level cues, we do not think that line terminators play a crucial role in crowding effects. It is true that the long gratings “remove” the terminators of the short or the gapped flankers, but terminators alone are insufficient to induce crowding in many other cases. For example, lines shorter than the target vernier lead to reduced crowding. Moreover, increasing the number of short lines reduces crowding (uncrowding), even though the number of terminators increases. In addition, as the authors themselves agree, in most of the uncrowding configurations used in other studies, neither the crowding nor uncrowding conditions have nearby line terminators (see Figures 5D and 5E).

Moore and Zheng's findings become easy to explain if one drops consideration of occlusion entirely. The LAMINART model explains their results as being due to the interaction of boundary connections between the target and flankers and a segmentation process that tries to isolate the contours of the target from the contours of the flankers. Their results fit within the model mechanisms that have already successfully explained other uncrowding phenomena. In conclusion, previous studies show strong evidence of mid-level mediation of crowding and even of grouping using 3D cues (Sayim et al., 2008). Thus, we agree with Moore and Zheng (2024) that if the Pacmans had led to the perception of a long grating, occluded by bars, uncrowding should have occurred. The reason for the absence of this effect is likely that the stimuli they used did not induce these percepts.

## Figures and Tables

**Figure 6.**
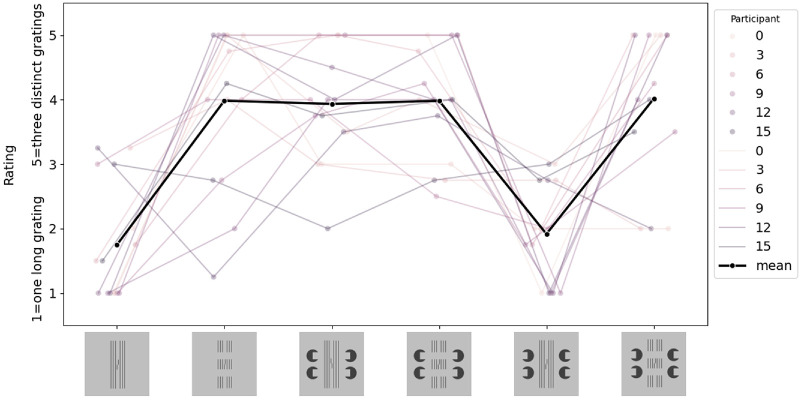
The complete ratings by all subjects, as an extension of Figure 2. The thicker black line indicates the mean rating. Each colored dot and line represent one subject.

## Appendix A. Appendix

### Instructions for the rating experiment

Instructions were presented in English or in French depending on which language the participant was more comfortable with.

(1) Here we want to test how people see overlapping elements. Please look at the display below and rate to what extent you perceive a rectangle on top of two circles using numbers from 1-5. 1 - “No, there are no overlapping elements” to 5 - “Yes, I see the square overlapping the circles.” Please give your answer using the number pad on the right side of the keyboard. [There followed an image of two Pacman style inducers facing each other, with an illusory rectangle between them.]
(2) The displays will appear in your peripheral vision. Only look at the fixation cross, never at the display directly. It will include a grating like the examples below. You have 2 Tasks: 1. First, while looking at the fixation cross, report the direction of the offset of the bottom line of the middle set of lines; Use the arrow keys (left and right). 2. Then you will rate whether, among all the other elements, the stimulus included 1 long grating or 3 separate gratings. (Press any key to continue) [There followed two images, one as shown in Figure 1C and one like in Figure 1D.]
(3) Please keep your eyes on the fixation cross and indicate in which direction you see the offset using the arrow keys. (Press any key to continue)
(4) Did you see 3 separate gratings or a single grating, partially covered by horizontal bars? Please indicate your answer with the numbers 1-5 on the number pad on the right side of the keyboard. 1 - “I saw a single grating” and 5 - “I saw 3 completely separate gratings”

Instructions 1 and 2 were shown once at the beginning of the experiment to introduce the task and establish the concept of responding to the rating task. Instructions 3 and 4 were shown before and after each trial.

### All participant responses

For completeness, Figure 6 shows the full rating responses given by all participants.
